# Cord Blood Acute Phase Reactants Predict Early Onset Neonatal Sepsis in Preterm Infants

**DOI:** 10.1371/journal.pone.0168677

**Published:** 2017-01-03

**Authors:** Leena B. Mithal, Hannah L. Palac, Ram Yogev, Linda M. Ernst, Karen K. Mestan

**Affiliations:** 1 Department of Pediatrics, Division of Infectious Diseases, Northwestern University Feinberg School of Medicine, Chicago, Illinois, United States of America; 2 Department of Preventive Medicine, Biostatistics Collaboration Center, Northwestern University Feinberg School of Medicine, Chicago, Illinois, United States of America; 3 Department of Pathology, Northwestern University Feinberg School of Medicine, Chicago, Illinois, United States of America; 4 Department of Pediatrics, Division of Neonatology, Northwestern University Feinberg School of Medicine, Chicago, Illinois, United States of America; Nanjing University Medical School Affiliated Nanjing Drum Tower Hospital, CHINA

## Abstract

**Background:**

Early onset sepsis (EOS) is a major cause of morbidity and mortality in preterm infants, yet diagnosis remains inadequate resulting in missed cases or prolonged empiric antibiotics with adverse consequences. Evaluation of acute phase reactant (APR) biomarkers in umbilical cord blood at birth may improve EOS detection in preterm infants with intrauterine infection.

**Methods:**

In this nested case-control study, infants (29.7 weeks gestation, IQR: 27.7–32.2) were identified from a longitudinal cohort with archived cord blood and placental histopathology. Patients were categorized using culture, laboratory, clinical, and antibiotic treatment data into sepsis groups: confirmed sepsis (cEOS, n = 12); presumed sepsis (PS, n = 30); and no sepsis (controls, n = 30). Nine APRs were measured in duplicate from cord blood using commercially available multiplex immunoassays (Bio-Plex Pro™). In addition, placental histopathologic data were linked to biomarker results.

**Results:**

cEOS organisms were *Escherichia coli*, *Streptococcus agalactiae*, *Proteus mirabilis*, *Haemophilus influenzae* and *Listeria monocytogenes*. C-reactive protein (CRP), serum amyloid A (SAA), haptoglobin (Hp), serum amyloid P and ferritin were significantly elevated in cEOS compared to controls (p<0.01). SAA, CRP, and Hp were elevated in cEOS but not in PS (p<0.01) and had AUCs of 99%, 96%, and 95% respectively in predicting cEOS. Regression analysis revealed robust associations of SAA, CRP, and Hp with EOS after adjustment for covariates. Procalcitonin, fibrinogen, α-2-macroglobulin and tissue plasminogen activator were not significantly different across groups. Placental acute inflammation was associated with APR elevation and was present in all cEOS, 9 PS, and 17 control infants.

**Conclusion:**

This study shows that certain APRs are elevated in cord blood of premature infants with EOS of intrauterine origin. SAA, CRP, and Hp at birth have potential diagnostic utility for risk stratification and identification of infants with EOS.

## Introduction

Despite advances in maternal screening and antibiotic prophylaxis, early onset sepsis (EOS) remains a major problem and causes infant morbidity and mortality. Of births in the US, 10% are preterm (<37 weeks gestation) resulting in 400,000 preterm neonates born yearly [1]. Invasive EOS disproportionately affects preterm infants with 47% of EOS cases and 92% of deaths from EOS occurring in this group [2–4]. A major gap in current medical care is the lack of reliability in identifying EOS, as perinatal risk factors and clinical signs are often nonspecific [5]. Current diagnostic tests for neonatal sepsis, such as blood count indices and serum C-reactive protein (CRP), have poor sensitivity and specificity and thus limited utility [6, 7]. Blood culture is the current gold standard for diagnosis of sepsis but the result is delayed and has a low sensitivity in neonates due to maternal antibiotic treatment, small specimen volumes, and low-grade bacteremia [8, 9]. Delayed diagnosis of sepsis and initiation of antibiotics increase morbidity and mortality [10]. As a result, broad-spectrum empiric antimicrobial treatment despite negative cultures is common in the preterm population causing serious adverse consequences including disrupted gastrointestinal and mucosal colonization, necrotizing enterocolitis, invasive fungal infections, and antibiotic resistance [11–13]. The “Choosing Wisely” campaign and antibiotic stewardship efforts highlight the need to avoid continuation of antibiotic therapy in asymptomatic infants without evidence of bacterial infection [14]. Development of novel, more reliable methods to diagnose sepsis in preterm infants is essential to improve their outcomes.

Umbilical cord blood evaluation is emerging as a potential means to aid in the diagnosis of neonatal pathology. Recent studies have focused on acute phase reactants (APR), cytokines, and adhesion molecules in peripheral blood as markers of infection and have shown significant associations with gestational age and poor neonatal outcomes [15–17]. Specifically with regard to infection, elevation of immune biomarkers in cord blood may facilitate earlier and more reliable detection of EOS. For example, cord blood procalcitonin (PCT) may be a useful parameter to detect early infected newborns [18–21]. However, small sample sizes, pooled data from term and preterm infants, and broad definitions of infection with few culture-proven EOS cases limit the utility of these studies. Currently no cord blood diagnostics for inflammation or infection are used in standard clinical practice.

Furthermore, intraamniotic infection, the primary route of infection in preterm infants with EOS, leads to inflammatory responses in the placenta [22]. Placental inflammation is associated with early onset neonatal infection [23–25], although only a fraction of infants with histologic chorioamnionitis develop EOS [22]. Some cord blood biomarker levels are associated with placental inflammation representing a neonatal response to a perinatal insult [26, 27]. Although the role of placental histopathology in the diagnosis of neonatal sepsis and treatment decisions remains unclear [28], placental inflammation may be a useful tool to identify infants exposed to intraamniotic infection.

The aim of the current study was to identify cord blood APRs associated with EOS in preterm infants. A secondary objective was to evaluate placental inflammation in EOS and in relation to cord blood APRs. We hypothesized that elevated biomarkers and placental inflammatory features on histopathology would be associated with EOS of intrauterine origin.

## Material and Methods

### Ethics statement

The study was approved by the Northwestern University Feinberg School of Medicine Institutional Review Board. Parental written informed consent was obtained for each patient prior to enrollment. Subsequent investigation was conducted according to the principles expressed in the Declaration of Helsinki.

### Study design

We conducted a nested case-control study of premature infants enrolled in the Prentice NICU Cord Blood Study, a longitudinal birth cohort established in 2008 at Prentice Women’s Hospital (Chicago, IL). Enrollment was dependent on parental consent and availability of cord blood. At the time of study, complete pertinent clinical data was available for 1,100 enrollees <37 weeks gestational age born between 2008 and 2014. Umbilical venous cord blood was collected at delivery into EDTA tubes and spun at 3,000rpm for 10 minutes in a refrigerated tabletop centrifuge to separate plasma, which was stored at -80°C until assay. Comprehensive clinical data of maternal and infant variables including demographic information, maternal risk factors, perinatal complications leading to preterm delivery, laboratory values, culture results, and antibiotics administered were recorded in the study database.

### Patient selection

**Fig 1:**
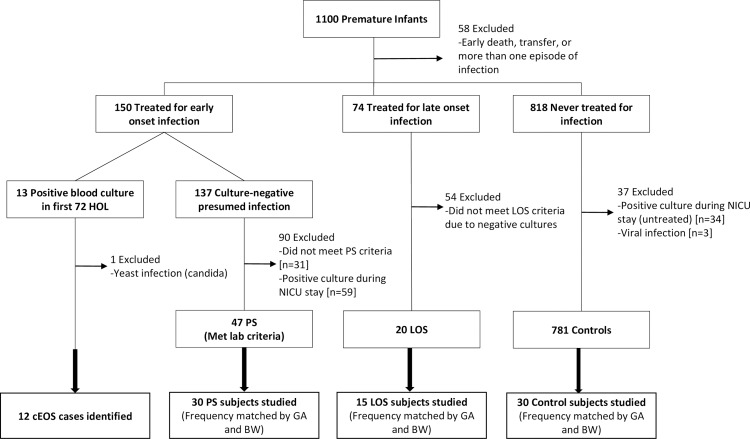
Study design and patient selection. Of 1100 enrolled infants, 12 had confirmed early onset sepsis (cEOS: positive blood culture with bacterial neonatal sepsis pathogen within 72 HOL and received antibiotic treatment ≥5 days). Presumed sepsis (PS) subjects were treated for early infection with antibiotics within first 72 HOL, had no positive sterile site culture during NICU stay, and had ≥2 abnormal lab criteria (↓ANC, ↑ I:T ratio, ↑CRP). Late onset sepsis (LOS) subjects had positive blood culture after 72 HOL treated with antibiotics. Control patients had no sterile site culture or antibiotic course >4 days throughout hospitalization. Patients with ≥2 infection episodes were excluded. PS, LOS and control patients were frequency matched to cEOS patients within target gestational age and birth weight ranges (±3 wks and ±400 gms). Infants were randomly selected from the group fitting GA/BW criteria.

Based on culture results, antibiotic treatment and laboratory data, 1,100 patients were classified by the following sepsis categories: confirmed EOS (cEOS), presumed EOS (PS), late onset sepsis (LOS), and controls according to pre-determined criteria based upon National Institute of Child Health and Human Development (NICHD) definitions for premature infants [29] (Fig 1). Infants were considered to have cEOS if a positive blood culture was present in the first 72 hours of life (HOL) with a single bacterial pathogen and they received an antibiotic course for ≥5 days. To maintain a stringent definition of cEOS for this study, patients with questionable true EOS pathogens, such as *Corynebacterium*, those with positive respiratory cultures only, and those with viral or fungal sepsis were not classified as confirmed EOS cases. Infants with PS were patients with no positive culture, but with ≥2 abnormal laboratory results from peripheral blood indicative of sepsis in the first 72 HOL and who received antibiotic treatment for ≥5 days at birth (Fig 1). Abnormal postnatal laboratory results were: CRP ≥1mg/dL [7], absolute neutrophil count outside normal range based on published guidelines according to gestational age [30], and elevated immature-to-total neutrophil ratio >0.2 [31]. Control patients had no positive culture throughout hospitalization and were never treated with >4 days of antibiotics (Table 1). The majority of control patients (24/30) received 2–3 days of antibiotics as “rule-out” of sepsis until blood culture was negative. PS and control patients were frequency matched based on gestational age and birth weight ranges in a 2:1 ratio to cEOS cases by gestational age (+/- 3 weeks) and birth weight (+/- 400 grams). In situations where more than one PS or control patient matched to the case, patients were randomly selected, masked to all clinical information, including laboratory values and placental histology.

**Table 1:** Case definition, demographics and placental histopathology.

Variable	Median (IQR) / N (%)			
	Control	cEOS	PS	Total Sample
	n = 30	n = 12	n = 30	N = 72
**Positive blood culture <72 HOL**	No	Yes	No	-
**Antibiotics initiated <72 HOL**	No	Yes	Yes	-
**Gestational age** (weeks)	29.9 (27.7–32.3)	30.2 (27.4–32.8)	29.6 (28.1–32.0)	29.7 (27.7–32.1)
**Birthweight** (g)	1365 (1055–1770)	1465 (1003–2051)	1360 (1000–1665)	1380 (1053–1760)
**Male gender**	12 (40)	8 (67)	24 (80)*	44 (61)
**Multiple gestation**	11 (37)	3 (25)	13 (43)	27 (38)
**Prolonged rupture of membranes**	8 (27)	8 (67)	11 (37)	27 (38)
**Preeclampsia**	6 (20)	0	7 (23)	13 (18)
**Clinical chorioamnionitis**	0	5 (42)*	4 (13)	9 (13)
**Placental histopathology**^b^				
Placental acute inflammation	17 (57)	12 (100)^**a**^	9 (32)	38 (54)
Maternal acute inflammation	16 (53)	12 (100)^**a**^	9 (32)	37 (53)
Fetal acute inflammation	6 (20)	12 (100)^**a**^	8 (29)*	26 (37)

Bonferroni adjusted p-values of 0.01 per test were used to determine statistical significance.

* Variable is significantly different from control group

^**a**^ Variable is significantly different from control and PS groups

^b^ Placental data not available for 2 PS patients (n = 28)

### Cord blood biomarkers

Nine APR proteins were measured in cord blood: PCT, CRP, haptoglobin (Hp), ferritin, fibrinogen, tissue plasminogen activator, serum amyloid A (SAA), serum amyloid P (SAP), and α-2-macroglobulin. These markers were selected upon careful review of the literature for potentially promising markers of EOS and available platforms from commercially available kits [7, 32–39]. The proteins were measured in cord blood plasma in duplicate using magnetic bead-based immunoassays (Bio-Plex Pro^TM^ Human Acute Phase Multiplex Assays [4-plex (α-2-macroglobulin, CRP, Hp, SAP) and 5-plex (ferritin, fibrinogen, PCT, SAA, tissue plasminogen activator)]; Bio-Rad Laboratories Inc.; Hercules, CA). Samples were thawed on ice and prepared in 1:1000 and 1:100 dilution for the respective panels. The kits were run on a Luminex 200 platform with Bio-Plex Manager 6.1 and Milliplex Analyst 5.1 software used for analysis. A 5 parameter logistic regression was used for standard curve fitting. Coefficient of variation for biomarkers were 0.53%-1.81%. The samples were prepared and assays were performed in the Comprehensive Metabolic Core at Northwestern University. Turnaround time to obtain results from this assay is 8 hours.

### Placental inflammation

Standardized gross and histologic analysis was performed on placentas from 84 of the 87 study patients, as part of the larger parent study protocols which have been previously described [16, 17]. Briefly, formalin-fixed paraffin-embedded sections stained with H&E were reviewed by a single perinatal pathologist who was masked to infant outcomes. For purposes of the present study, the presence of acute inflammation (AI) was recorded and subcategorized as maternal and or fetal AI, and graded by stage (1–3) according to distinct histologic lesions [40, 41]. Maternal AI was identified by neutrophil infiltration of chorion (stage 1), amnion (stage 2), and necrotizing chorioamnionitis (stage 3). Fetal AI was identified by neutrophil diapedesis through the wall of the chorionic vessels or umbilical vein (stage 1), umbilical artery (stage 2), and necrotizing funisitis (stage 3) defined by neutrophil karyorrhexis in a band-like configuration within Wharton’s jelly [41].

### Statistical analysis

Statistical analysis was performed in collaboration with Northwestern Feinberg School of Medicine Biostatistics Collaboration Center using SAS® software, version 9.4 (Cary, NC) and STATA® software, version 13.1 (College Station, TX). Sample characteristics were reported as medians (25^th^-75^th^ interquartile range; IQR) or frequencies and percentages. The Kruskal-Wallis tests and chi-square or Fisher’s exact tests were used to identify differences in variables across sepsis categories as appropriate. For variables showing statistical significance, comparisons of sepsis categories were assessed using the Wilcoxon rank sum test and chi-square/Fisher’s exact tests. Bonferroni-adjusted p-values of p <0.01 were used to account for multiple comparisons (5 individual comparisons between sepsis groups, including a late onset sepsis group in original study design).

Logistic regression modeling was used to further quantify the relationship between each biomarker and cEOS with true controls serving as the reference group. First, bivariate models were used to evaluate the unadjusted associations between cEOS and each biomarker separately. Multivariable logistic regression was then used to evaluate the associations between biomarker and cEOS when controlling for presence of fetal AI. Fetal AI was used to represent the placental AI variables to avoid potential multicollinearity between inflammatory markers. Receiver operating characteristic (ROC) curves and associated area under the curves (AUCs) were generated for all nine cord blood APRs to further evaluate diagnostic accuracy for cEOS and explore optimal cutoff values. In a separate sensitivity analysis, outlying values for each biomarker identified using leverage plots and DFbeta values greater than 2 in absolute value were omitted.

The effect of sepsis on SAA, CRP, Hp, ferritin, and SAP was further analyzed using multiple linear regression with all biomarkers log transformed prior to modeling. To build multivariable models for each biomarker, first all potential covariates, including: gestational age, birthweight, gender, multiple gestation, PROM, preeclampsia, clinical chorioamnionitis, any placental AI, fetal AI, and maternal AI were assessed in unadjusted models. Using a model entry criterion of p ≤ 0.10, each potential predictor was entered into each model and a manual backward selection procedure was used to determine a final model for each biomarker.

## Results

### Demographics

The 72 patients presented here (groups: cEOS, PS and controls) had a median gestational age of 29.7 weeks (IQR: 27.7–32.2) and median birth weight of 1380g (IQR: 1053-1760g). Characteristics of cEOS, PS, and control patients were similar with no significant differences in gestational age, birth weight, multiple gestation, prolonged rupture of membranes (PROM), and preeclampsia between groups (Table 1; S1 Table). No control patients had clinical chorioamnionitis, which was significantly different from cEOS patients. In the twelve cEOS patients, the organisms isolated in postnatal blood culture were *Escherichia coli* (n = 7), *Streptococcus agalactiae* (n = 2), *Proteus mirabilis* (n = 1), *Haemophilus influenzae* (n = 1) and *Listeria monocytogenes* (n = 1).

### Cord blood acute phase reactants

**Fig 2:**
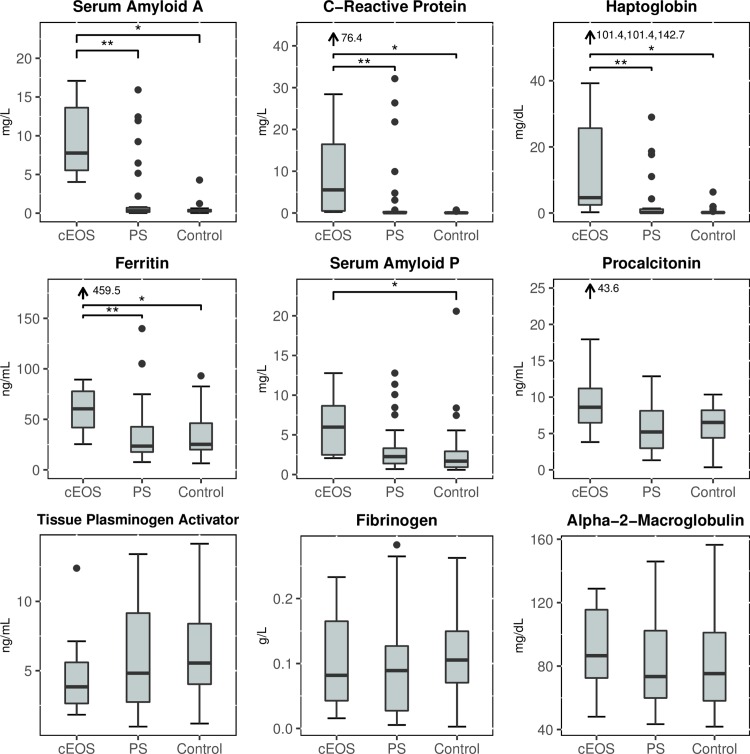
Acute phase reactant levels in sepsis groups. Box and whisker plots displaying distribution of 9 APR biomarkers (SAA, CRP, Hp, ferritin, SAP, PCT, tissue plasminogen activator, fibrinogen, α-2-macroglobulin) in each sepsis category. *Indicates significant difference in APR values between cEOS and control groups. **Indicates significant difference in APR values between cEOS and PS groups.

The cEOS group had significantly elevated levels of five biomarkers compared to the control group: CRP, SAA, Hp, SAP, and ferritin (all p<0.01, Table 2, Fig 2, S1 Table). The APR levels in the cEOS group were also elevated compared to the PS group for 4 biomarkers: CRP, SAA, Hp, and ferritin. In contrast, APR levels in the PS group were not significantly different compared to the control group (Table 2, Fig 2, S1 Table). Tissue plasminogen activator, fibrinogen, and α-2-macroglobulin levels were not significantly difference between the three groups. PCT was elevated in all groups, and while it was elevated in the cEOS group, did not reach a statistically significant difference compared to the control (p = 0.03) or the PS (p = 0.03) groups using our adjusted significance threshold of p<0.01 per test.

**Table 2:** Cord blood acute phase reactants: comparison of cEOS to other sepsis groups and controls.

Acute Phase Reactant	Median (IQR)			p-value^a^
	Control	cEOS	PS	
	n = 30	n = 12	n = 30	
**SAA** (mg/L)	0.3 (0.2–0.5)*	7.8 (5.5–14.1)	0.3 (0.1–0.8)*	**< .0001**
**CRP** (mg/L)	<0.1 (<0.1–0.1)*	5.5 (0.4–19.5)	0.1 (<0.1–0.3)*	**< .0001**
**Hp** (mg/dl)	0.2 (0.1–0.2)*	4.7 (2.4–30.2)	0.2 (0.1–1.2)*	**< .0001**
**Ferritin** (ng/ml)	25.2 (20.0–46.5)*	60.4 (40.1–79.5)	23.5 (17.4–46.2)*	**0.002**
**SAP** (mg/L)	1.7 (0.9–2.9)*	6 (2.5–9.9)	2.3 (1.4–3.4)	**0.005**
**PCT** (ng/ml)	6.5 (4.2–8.3)	8.6 (6.2–12)	5.2 (2.8–8.2)	0.033
**TPA** (ng/ml)	5.5 (4–8.7)	3.8 (2.6–5.7)	4.8 (2.6–9.3)	0.272
**Fibrinogen** (g/L)	0.1 (0.1–0.2)	0.1 (0–0.2)	0.1 (0–0.1)	0.514
**α-2-macroglobulin** (mg/dl)	75.3 (57.8–101.8)	86.6 (71.6–116.2)	73.4 (59.8–102.7)	0.483

*Significantly different from cEOS group. Significance was based on pairwise comparisons using Wilcoxon rank sum test. Bonferroni-adjusted p-values of 0.01 per test were used to account for multiple comparisons.

^a^p-values for Kruskal-Wallis test.

### Placental inflammation

**Fig 3:**
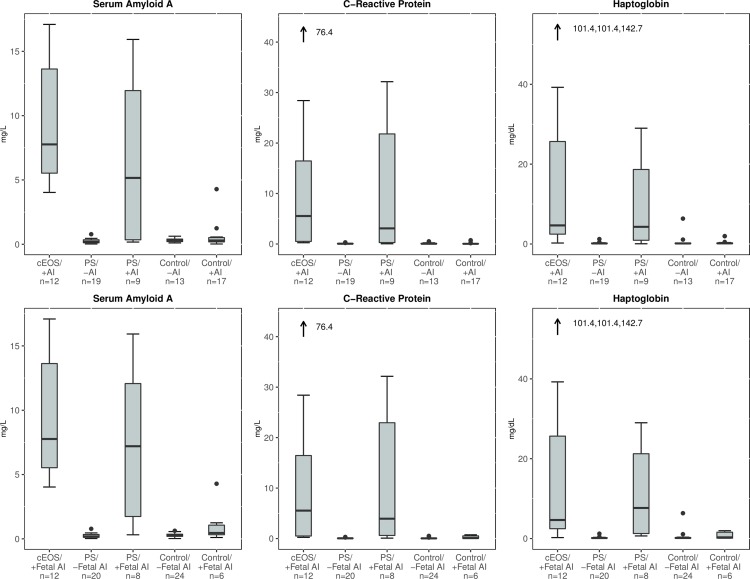
Serum amyloid A, C-reactive protein, and haptoglobin levels in sepsis groups by AI. Box and whisker plots displaying distribution of 3 APR biomarkers (SAA, CRP, Hp) in each sepsis category with and without fetal AI [bottom row] and any placental AI (maternal and/or fetal) [top row].

AI on placental pathology including maternal, fetal, maternal/fetal high-stage (II-III), and funisitis was each significantly more prevalent in cEOS than both PS and control groups (Chi-square, p<0.01). In fact, all cEOS patients had maternal and fetal side AI. Twenty-nine percent of PS and 20% of controls also had fetal AI. Maternal inflammation was present in 32% of PS patients and 53% of controls (Table 1). Pairwise comparisons were repeated in only the patients with fetal AI (including 12/12 cEOS and 8/28 PS patients). The biomarker levels were not significantly different between cEOS and PS patients with fetal AI. Infants in the control group with placental AI did not have significantly elevated APR levels compared to control patients without placental AI (p-values ranging 0.17–0.99) (Fig 3, individual comparisons for each APR in S2 Table).

### Regression analysis

Significant bivariate associations for cEOS were present in logistic regression models for ferritin, PCT, SAA, Hp, CRP, and SAP, PROM, clinical chorioamnionitis (chorio), and fetal AI. When adjusting for fetal AI, the association between cEOS and each biomarker was attenuated with SAA as the only remaining significant marker (p = 0.037). However, this analysis is limited by the presence of fetal AI in 100% of cEOS patients. Potential outlying observations for ferritin (459 ng/ml), Hp (101.4 and 142.7 mg/dL), and SAP (20.6 mg/L) were identified and excluded in a sensitivity analysis. Overall significance and interpretation of results were unchanged.

**Fig 4:**
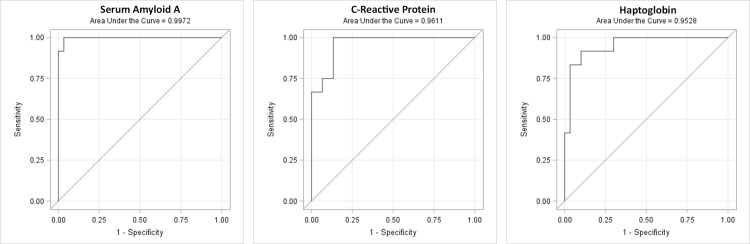
Receiver operating characteristic curves. ROC curves for serum amyloid A, C-reactive protein, and haptoglobin. The above acute phase reactants had areas under the curve of >95% for prediction of EOS.

ROC curves were generated for each of the 9 biomarkers individually. Areas under the curve (AUCs) with high values were 99%, 96%, and 95% for SAA, CRP, and Hp, respectively with proposed cut-off points of 4 mg/L, 0.25 mg/L, and 1.1 mg/dl (Table 3, Fig 4). The cut-off values were chosen at the point that minimizes the distance from the sensitivity = 0, 1-specificity = 1 point (upper-left corner of ROC curve; perfect point method).

**Table 3:** Receiver operating characteristic analysis: AUC and biomarker cut-offs.

Acute Phase Reactant	AUC	Cut-off value
**SAA** (mg/L)	99.7%	4.0 mg/L
	(95% CI: 99.0–100.0)	
**CRP** (mg/L)	96.1%	0.25 mg/L
	(95% CI: 91.1–100.0)	
**Hp** (mg/dl)	95.3%	1.1 mg/dl
	(95% CI: 89.0–100.0)	
**Ferritin** (ng/ml)	82.5%	51 ng/ml
	(95% CI: 69.8–95.2)	
**SAP** (mg/L)	80.0%	2.5 mg/L
	(95% CI: 66.4–93.6)	
**PCT** (ng/ml)	73.1%	-
	(95% CI: 54.1–92.0)	
**TPA** (ng/ml)	66.7%	-
	(95% CI: 47.7–85.7)	
**α-2-macroglobulin** (mg/dl)	59.2%	-
	(95% CI: 40.9–77.5)	
**Fibrinogen** (g/L)	57.2%	-
	(95% CI: 34.7–79.8)	

Results of receiver operating characteristic curve analysis for APRs for prediction of early onset sepsis. AUC >90% indicates significant potential utility of a test for diagnosis of sepsis. Cut-off values were determined by perfect point (0, 1) method for biomarkers with AUC > 80%.

Consistent with previous analyses, linear regression modeling of sepsis demonstrated significant beta coefficients in crude models for Hp, CRP and SAA with cEOS (p<0.001) and for SAP and ferritin (p<0.01) as shown in Table 4. The associations between cEOS and ferritin or SAP were no longer significant when adjusting the ferritin model for clinical chorioamnionitis and fetal AI and the SAP model for fetal AI. However, the associations between sepsis and Hp, CRP, and SAA remained significant after adjustment for relevant covariates included in final multiple linear regression models (p-values of 0.029, 0.012, and 0.001 respectively).

**Table 4:** Linear regression model for sepsis effect on acute phase reactants.

Acute Phase Reactant^a^	Unadjusted		Adjusted^b^		Covariates in Adjusted Model
	β (95% CI)	p-value	β (95% CI)	p-value	
**SAA**					
cEOS	3.34 (2.45–4.22)	**< .0001**	1.35 (0.57–2.12)	**0.001**	Gestational age, Chorio, Fetal AI
PS	0.4 (-0.27–1.07)	0.236	-0.04 (-0.52–0.44)	0.870	
Control	[Ref]		[Ref]		
**CRP**					
cEOS	4.04 (2.78–5.3)	**< .0001**	1.52 (0.35–2.69)	**0.012**	Mult gestation, Chorio, Fetal AI
PS	1.2 (0.25–2.16)	**0.014**	0.66 (-0.06–1.38)	0.070	
Control	[Ref]		[Ref]		
**Hp**					
cEOS	3.61 (2.44–4.78)	**< .0001**	1.31 (0.14–2.48)	**0.029**	Chorio, Fetal AI
PS	0.88 (-0.01–1.76)	0.0530	0.4 (-0.32–1.12)	0.2750	
Control	[Ref]		[Ref]		
**Ferritin**					
cEOS	0.8 (0.32–1.28)	**0.001**	0.15 (-0.39–0.69)	0.577	Chorio, Fetal AI
PS	-0.06 (-0.42–0.3)	0.737	-0.28 (-0.61–0.05)	0.092	
Control	[Ref]		[Ref]		
**SAP**					
cEOS	0.99 (0.44–1.55)	**0.0007**	0.35 (-0.29–0.98)	0.283	Fetal AI
PS	0.26 (-0.16–0.68)	0.219	0.15 (-0.25–0.55)	0.444	
Control	[Ref]		[Ref]		

^a^ Biomarkers were log transformed for all linear regression modeling.

^b^ Final adjusted models are as follows

(log) SAA = -4.06 + 1.35*cEOS + -0.04*PS + 0.08*Gestational age + 1.47*Chorioamnionitis + 1.71*Fetal AI

(log) CRP = -3.02 + 1.52*cEOS + 0.66*PS + -0.58*Multiple gestation + 2.23*Chorioamnionitis + 1.91*Fetal AI

(log) Hp = -2.05 + 1.31*cEOS + 0.40*PS + 1.54*Chorioamnionitis + 2.07*Fetal AI

(log) Ferritin = 3.28 + 0.15*cEOS + -0.28*PS + 0.89*Chorioamnionitis + 0.35*Fetal AI

(log) SAP = 0.47 + 0.35*cEOS + 0.15*PS + 0.81*Fetal AI

## Discussion

The use of novel cord blood biomarkers for early and more reliable detection of EOS would address an important clinical challenge. In this study of cord blood APRs in preterm infants, we found that three proteins SAA, CRP, Hp were significantly elevated in cases of cEOS compared to controls and patients with PS. These APRs demonstrated strong association with cEOS in regression analyses and demonstrated high AUCs in ROC analysis. Our results suggest that these APRs could be useful for detection and exclusion of EOS in preterm infants. It is notable that the PS group, as a whole, had a similar biomarker profile to the control group who were not treated for sepsis, different from the cEOS group. As demonstrated by Fig 2 however, there were certain PS patients with elevated cord blood SAA, CRP and Hp (n = 6) similar to cEOS patients, and these PS patients also had histologic evidence of placental AI. In fact, the stratified analysis by fetal AI showed no significant differences between PS patients with fetal AI and the cEOS patients. One possible explanation is that these PS patients were treated for infection that was missed by blood culture. The other PS patients that did not have elevated biomarkers probably did not have an early infection and instead were overtreated with an empiric antibiotic course due to nonspecific clinical and laboratory abnormalities. Thus, distinguishing patients with true EOS by increased cord blood APR levels could help guide antibiotic management decisions and reduce unnecessary antibiotic use. Placental AI is associated with EOS and is present in the majority of patients with biomarker elevation. The possibility exists that biomarker elevation could reflect placental inflammation. However, there were many control patients and PS patients with placental AI that did not have elevated APRs. There were also no significant APR differences in the controls with and without placental AI (Fig 3, S2 Table). Further study and analysis of cord blood APRs with a larger preterm infant population including cases of EOS with and without placental inflammation would help to clarify these specific associations.

There are no established normal ranges for fibrinogen, tissue plasminogen activator, and α-2-macroglobulin in cord blood of preterm infants. The levels of tissue plasminogen activator found in our three groups were similar to those reported as normal in peripheral serum of children [42]. Fibrinogen and α-2-macroglobulin levels in all three groups were generally lower than reported values in premature infant serum and healthy term infant cord blood respectively [43, 44]. This study cohort also had lower cord blood ferritin levels than previously reported (<5^th^ percentile value <35 ng/ml in preterm infants whereas our median/IQR range was 30.2 ng/ml (19–51)) [45].

A few studies have shown that cord blood PCT may have the greatest potential to predict EOS with reported sensitivity of 75–88% and specificity 69–99% [18]. In contrast, cord blood CRP results have widely varied ranges (sensitivity 25–74% and specificity 22–97%), and thus CRP has not been considered a promising cord blood biomarker [18]. The validity of these existing studies is limited by small sample size, low numbers of culture-positive sepsis, and inclusion of both term and preterm infants. In the current study, PCT levels were elevated in all sepsis groups (median 6.5 ng/ml IQR: 4.2–8.3) compared to previously published normal cord blood ranges (<0.5 ng/ml to 1.22 ng/ml [19–21]). In addition, the elevated PCT level in cEOS compared to PS and control groups, did not reach statistical significance in contrast to the results in a previous study [19]. Thus, cord blood PCT may not be a helpful diagnostic tool for cEOS in preterm infants.

Our results suggest that cord blood SAA, CRP, and Hp are very likely to perform well and provide clinical utility for EOS diagnosis. It is important to note that the cut-off values are notably lower than the normal published cut-offs values in the peripheral postnatal serum (e.g., CRP cut-off of 0.25 mg/L in our study compared to published cut-off of 10 mg/L) [7]. Our lower levels are supported by a study of cord blood SAA and CRP in normal, term infants that reported a median CRP of 0.19 mg/L and SAA of 5.86 mg/L [46]. Thus, the lower normal range cut-off for cord blood CRP and SAA of preterm infants reported in our study may lead to improved sensitivity for EOS. Despite increasing literature on SAA as a marker of sepsis, this is the first study of SAA in cord blood of preterm infants with EOS. Our findings also corroborate results from Buhimschi and colleagues who reported Hp "switch-on pattern” in preterm infants with EOS based on proteomics mapping [37]. Interestingly, SAA, CRP, and Hp are all acute phase proteins of hepatic origin, downstream of IL-6 in the JAK/STAT3 pathway and may indicate the importance of hepatocellular inflammatory response in early neonatal infection. Further study on cord blood markers may not only led to improved diagnostics, but also provide insight into the pathophysiology of perinatal infection in preterm infants and potential therapeutic targets.

The role of placental histopathologic inflammation in diagnosis of early onset neonatal infection has not been established. In our study, all cases of cEOS were found to have histologic evidence of maternal and fetal AI suggesting that all cEOS patients likely had intrauterine infection rather than intrapartum acquisition of pathogens at birth. This observation is consistent with previous reports that placental histology is a sensitive marker of intraamniotic infection associated with EOS. Yet, 20–50% of non-EOS infants (including controls) had fetal or maternal AI. Therefore, while placental histology is a sensitive marker for predicting EOS in preterm infants with intrauterine infection, it is not specific. This underscores the importance of adjunct markers that will improve the specificity of the diagnostic tests and with a high positive predictive value. Our findings support that some cord blood APRs may serve in this capacity. Again, more studies, including EOS without intrauterine inflammation, are needed to further elucidate the interplay between placenta, cord blood biomarkers, and EOS.

We have reported cEOS, PS and “clean” controls in these results, none of which had LOS, to eliminate potential confounding from LOS. We did, however, have a fourth group of patients with LOS who were not treated for EOS, serving as a secondary control group. SAA, CRP, Hp, SAP, and PCT were elevated in cEOS compared to LOS patients, but there were no significant differences in the cord blood APR profile of LOS compared to controls (S3 Table). The analysis of this group also supports the hypothesis that the cord blood APR profile is distinct in patients with EOS, not related to an infection after 72 hours of life.

While our study is limited by the relatively small sample size and the few APRs tested, our stringent definition of sepsis allowed us to focus on the primary endpoint of confirmed sepsis using the current diagnostic “gold standard” (positive blood culture within 72 hours of birth) without confounding by clinically diagnosed sepsis. Alternate molecular methods of pathogen detection, such as 16S rRNA PCR, are being studied and may serve as a sensitive adjunct to culture results in the future, but further evidence is required for PCR-based methods to be incorporated into clinical standard of care [47]. Our distinction between PS and culture-proven cEOS provided a high-quality analysis of APRs as a screening tool for infection. Although the number of cEOS patients was small, it is consistent with reported rates of 1–2% [3]. Other limitations include the retrospective nature of the sepsis definitions and inclusion of preterm infants from only a single NICU. Yet, restricting our inclusion to only preterm infants allowed us to focus our analysis on a subpopulation of neonates with the highest risk of EOS consequences that are likely to have distinct pathophysiology of EOS than many full term infants.

The results of the current study provide an important foundation for generating new hypotheses and novel approaches using cord blood APRs as diagnostic indicators of EOS. However, these findings need to be verified by prospective multi-center studies with clinical decision-making driven by interpretation of these biomarkers and longitudinal follow-up of outcomes. Such studies would provide the data required to incorporate cord blood diagnostics into standard of care practice.

In conclusion, cord blood APRs seem to be sensitive markers for identification of infants with EOS and for ruling out sepsis, as they reflect the fetal state of inflammation in the intrauterine environment that may lead to early neonatal infections in premature infants. The study supports the role of APR proteins in cord blood (in particular: SAA, CRP, and Hp) for early detection and risk stratification of infants for EOS. Intrauterine inflammatory processes in the placenta appear to be associated with EOS and changes in cord blood APRs. Thus, cord blood biomarker levels with consideration of placental histopathology may improve the overall management of EOS in preterm infants.

## Supporting Information

S1 Table Pairwise comparisons of sample characteristics and acute phase reactants between sepsis groups. (DOCX)

S2 Table APR values by placental and fetal acute inflammation in control patients (n = 30) (DOCX)

S3 Table Late onset sepsis group characteristics, placental inflammation, and acute phase reactants. (DOCX)

S4 Table Database with subject demographics, culture results, placental characteristics, and acute phase reactants. (XLSX)
