# Supplementary material for: Cord Blood Acute Phase Reactants Predict Early Onset Neonatal Sepsis in Preterm Infants
Source: PLoS One. 2017 Jan 3;12(1):e0168677. doi: 10.1371/journal.pone.0168677 (PMC5207723; doi:10.1371/journal.pone.0168677)
Supplement: S1 Table — (DOCX) [file pone.0168677.s001.docx]

**S1 Table. Pairwise comparisons for sample characteristics and acute phase reactants between sepsis groups.**

| **Variable**^a^ | **Comparison (p-values**^b^**)** | | |
| --- | --- | --- | --- |
|  | **cEOS vs Control** | **cEOS vs PS** | **PS vs Control** |
| **Gestational age** (weeks) | 0.8899 | 0.8248 | 0.9179 |
| **Birthweight** (g) | 0.7715 | 0.5527 | 0.7075 |
| **Gender** | 0.1180 | 0.4331 | **0.0016** |
| **Clinical chorioamnionitis** | **0.0009** | 0.0900 | 0.1124 |
| **Placental histopathology** |  | | |
| Fetal acute inflammation | **<.0001** | **<.0001** | 0.4459 |
| Maternal acute inflammation | **0.0034** | **<.0001** | 0.1034 |
| Any inflammation (acute or chronic) | **0.0077** | **<.0001** | 0.0606 |
| **PCT** (ng/ml) | 0.0268 | 0.0259 | 0.4109 |
| **SAA** (mg/L) | **<.0001** | **0.0004** | 0.9413 |
| **CRP** (mg/L) | **<.0001** | **0.0015** | 0.0209 |
| **Hp** (mg/dL) | **<.0001** | **0.0017** | 0.1466 |
| **Ferritin** (ng/ml) | **0.0023** | **0.0034** | 0.5516 |
| **SAP** (mg/L) | **0.0047** | 0.0168 | 0.1909 |

^a^ Variables included were those significant when compared across groups using the Kruskal-Wallis test for continuous variables and chi-square or Fisher's exact tests for categorical variables

^b^ Tests were conducted using Bonferroni adjusted p-values of 0.01 per test.
