# Supplementary material for: Cord Blood Acute Phase Reactants Predict Early Onset Neonatal Sepsis in Preterm Infants
Source: PLoS One. 2017 Jan 3;12(1):e0168677. doi: 10.1371/journal.pone.0168677 (PMC5207723; doi:10.1371/journal.pone.0168677)
Supplement: S2 Table — (DOCX) [file pone.0168677.s002.docx]

**S2 Table: APR values by fetal and acute inflammation in control patients** (n=30)

| **Acute Phase Reactant** | **+Fetal AI** | **-Fetal AI** | **p-value*** | **+AI** | **-AI** | **p-value*** |
| --- | --- | --- | --- | --- | --- | --- |
|  | **n=6** | **n=24** |  | **n=17** | **n=13** |  |
| **SAA** (mg/L) | 0.5 (0.3-1.2) | 0.3 (0.2-0.4) | 0.1369 | 0.3 (0.2-0.5) | 0.3 (0.2-0.4) | 0.9999 |
| **CRP** (mg/L) | 0.1 (0-0.7) | 0 (0-0.1) | 0.2741 | 0 (0-0.1) | 0 (0-0.1) | 0.7716 |
| **Hp** (mg/dl) | 0.3 (0.1-2) | 0.1 (0.1-0.2) | 0.3993 | 0.2 (0.1-0.3) | 0.1 (0.1-0.2) | 0.6787 |
| **Ferritin** (ng/ml) | 35.2 (20-46.5) | 25.2 (19.5-43.4) | 0.8172 | 30.6 (19-49.2) | 22.6 (20.4-30.9) | 0.5351 |
| **SAP** (mg/L) | 3.3 (1.6-3.9) | 1.3 (0.8-2.7) | 0.1027 | 1.5 (0.9-2.8) | 2.3 (1-4.2) | 0.5216 |
| **PCT (**ng/ml) | 8.9 (5.3-9.6) | 6.3 (4.2-7.7) | 0.0840 | 7.5 (5.3-8.9) | 6.2 (4.1-7.3) | 0.1713 |
| **TPA** (ng/ml) | 8.6 (5.2-13.6) | 5.1 (3.9-7.3) | 0.2327 | 5.5 (3.8-11.6) | 5.7 (4-7.1) | 0.8845 |
| **Fibrinogen** (g/L) | 0.2 (0.1-0.2) | 0.1 (0.1-0.1) | 0.1965 | 0.1 (0.1-0.2) | 0.1 (0.1-0.1) | 0.8357 |
| **α2M** (mg/dl) | 99.5 (59.1-135.7) | 73.9 (52.7-98.9) | 0.2529 | 74.6 (59.1-99.2) | 87.1 (50.1-101.8) | 0.9010 |

*p-values for Wilcoxon rank sum test
