# Supplementary material for: Cord Blood Acute Phase Reactants Predict Early Onset Neonatal Sepsis in Preterm Infants
Source: PLoS One. 2017 Jan 3;12(1):e0168677. doi: 10.1371/journal.pone.0168677 (PMC5207723; doi:10.1371/journal.pone.0168677)
Supplement: S3 Table — (DOCX) [file pone.0168677.s003.docx]

**S3 Table. Late onset sepsis group characteristics, placental inflammation, and acute phase reactants.**

| **Variable** | **LOS**  n=15 | **Pairwise comparisons**  p-value^a^ | |
| --- | --- | --- | --- |
|  | **Median (IQR) / N (%)** | **LOS vs Control**^b^ | **LOS vs cEOS**^b^ |
| **Gestational age** (weeks) | 28.3 (26.1-29.3) | 0.0170 | 0.0412 |
| **Birthweight** (g) | 1075 (875-1155) | **0.0096** | 0.0392 |
| **Male gender** | 9 (60) | 0.2049 | 0.9999 |
| **Clinical chorioamnionitis** | 0 | N/A^c^ | **0.0098** |
| **Placental histopathology** (n=14) |  | | |
| Acute inflammation (AI) | 7 (50) | 0.6791 | **0.0064** |
| Maternal AI | 7 (50) | 0.8366 | **0.0064** |
| Fetal AI | 5 (37) | 0.2867 | **0.0007** |
| **Acute phase reactants** |  | | |
| **PCT** (ng/ml) | 4.6 (4.1-6.1) | 0.0397 | **0.0066** |
| **SAA** (mg/L) | 0.3 (0.3-0.4) | 0.5501 | **0.0002** |
| **CRP** (mg/L) | <0.1 (<0.1-0.1) | 0.5113 | **0.0004** |
| **Hp** (mg/dl) | 0.2 (0.1-0.4) | 0.8858 | **0.0009** |
| **Ferritin** (ng/ml) | 35.2 (13.3-51.0) | 0.8764 | 0.0286 |
| **SAP** (mg/L) | 1.4 (1.1-4.4) | 0.7832 | **0.0084** |
| **α-2-macroglobulin** (mg/dl) | 74.8 (60-89.8) | 0.8388 | 0.2620 |
| **TPA** (ng/ml) | 6.8 (4.6-8.5) | 0.3778 | 0.0649 |
| **Fibrinogen** (g/L) | 0.1 (<0.1-0.2) | 0.5424 | 0.7905 |

^a^ Variables included were those significant when compared across groups using the Kruskal-Wallis test for continuous variables and chi-square or Fisher's exact tests for categorical variables

^b^ Tests were conducted using Bonferroni adjusted p-values of 0.01 per test (0.05/5).

^c^ This comparison could not be evaluated due to no presence of chorioamnionitis in both groups.
